# Utilization of aorta-specific fat attenuation index in evaluating disease activity and inflammation in Takayasu arteritis

**DOI:** 10.1186/s13244-026-02237-0

**Published:** 2026-03-30

**Authors:** Zhijie Jian, Hui Zhang, Hui Liu, Jianqing Sun, Jian Yang, Qi An, Jing Luo, Lele Cheng

**Affiliations:** 1 Department of Radiology, The First Affiliated Hospital of Xi’an Jiaotong University, 710061 Xi’an, People’s Republic of China; 2 Biobank, The First Affiliated Hospital of Xi’an Jiaotong University, 710061 Xi’an, People’s Republic of China; 3 Shanghai United Imaging Advanced Technology Research Institute Co., Ltd., No. 2258, Chengbei Road, JiaDing District, Shanghai, People’s Republic of China; 4 Department of Rheumatology and Immunology, The First Affiliated Hospital of Xi’an Jiaotong University, 710061 Xi’an, People’s Republic of China

**Keywords:** Takayasu arteritis, Perivascular adipose tissue (PVAT), Computed tomographic angiography (CTA), Fat attenuation index (FAI), Aorta

## Abstract

**Objectives:**

Takayasu arteritis (TA) is a rare vasculitis impacting the aorta and its branches in young people, leading to severe health issues and early death. Accurate assessment of disease activity is crucial for treatment. Chronic vascular wall inflammation is a key feature of TA. The peri-coronary fat attenuation index (FAI), measured via coronary computed tomographic angiography (CTA), is a new marker for coronary inflammation and cardiovascular risk. However, the value of aorta-specific FAI for diagnosing and assessing TA activity is still uncertain.

**Materials and methods:**

The study examined 101 TA patients and 77 matched controls with aortic CTA from September 2015 to May 2023. Disease activity was assessed using the Kerr score, and perivascular fat was analyzed with semi-automatic software. Aortic CTA parameters included maximal wall thickness, affected branches, luminal abnormalities, standardized CT values, and collateral branches. The primary focus was on the link between aorta-specific FAI and TA activity.

**Results:**

Aorta-specific FAI was higher in active TA patients than inactive ones, with medians of −75.51 HU and −80.53 HU, respectively (*p* = 0.002). FAI positively correlated with the Kerr score, aortic wall thickness, and erythrocyte sedimentation rate. A one-unit FAI increase raised TA activity odds by 14% (OR = 1.14, *p* = 0.025). Combining FAI with other imaging parameters improved TA activity diagnosis, reaching an AUC of 0.80.

**Conclusion:**

Aorta-specific FAI from CTA can assess TA activity, enhance morphological diagnosis and improve vessel wall inflammation evaluation.

**Critical relevance statement:**

This study demonstrates that the aorta-specific FAI derived from routine CTA provides an objective, quantifiable measure of vascular inflammation and disease activity in TA, enhancing the diagnostic yield of standard morphological assessment.

**Key Points:**

- Accurate assessment of disease activity in Takayasu arteritis remains challenging with current clinical and standard imaging criteria.
- Aorta-specific fat attenuation index is significantly elevated in active Takayasu arteritis and correlates with established markers of inflammation and vascular wall thickening.
- Combining fat attenuation index with CTA morphology enhances non-invasive diagnosis of active Takayasu arteritis, without extra cost or radiation.

**Graphical Abstract:**

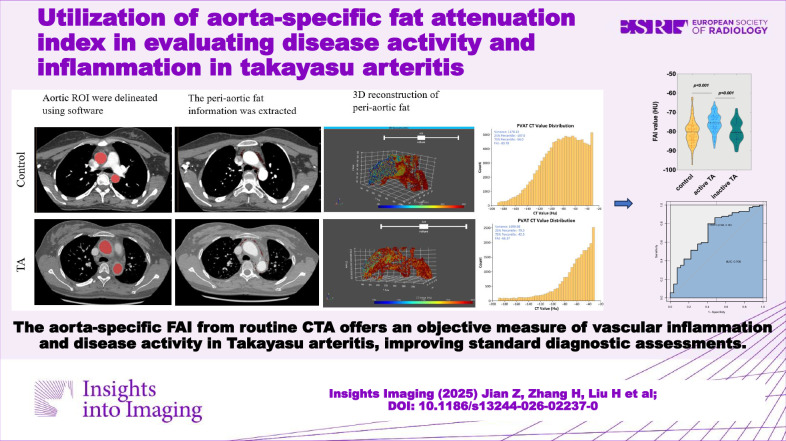

## Introduction

Takayasu arteritis (TA) is an uncommon type of granulomatous vasculitis predominantly affecting the aorta and its major branches, resulting in considerable morbidity and premature mortality [1, 2]. Effective management of vascular inflammation is essential for successful treatment [3, 4]. Concurrently, monitoring disease activity during pharmacotherapy is critical for assessing therapeutic efficacy and adjusting treatment regimens, thereby improving patient outcomes and reducing healthcare expenditures. Clinically, assessment of TA activity primarily relies on scoring scales of clinical symptoms and nonspecific inflammatory biomarkers, such as erythrocyte sedimentation rate (ESR) and C-reactive protein (CRP). However, this approach can introduce ambiguity and subjectivity. These considerations highlight the necessity for a comprehensive and cost-effective strategy that integrates both disease severity and activity assessment.

Although non-invasive imaging modalities such as computed tomography angiography (CTA) and magnetic resonance angiography (MRA) have been employed for evaluating wall thickening and luminal stenosis in affected blood vessels, they do not provide a precise assessment of disease activity [4]. The use of 18F-fluoro-D-glucose positron emission tomography (PET)-computed tomography (CT) can be a valuable tool in characterizing disease activity in TA, but its application is limited by high cost and radiation exposure [5]. CTA, as a routine imaging examination method for blood vessels, is both convenient and non-invasive. If more information can be obtained from CTA images, it has high cost-effectiveness and feasibility for patients and clinical practice.

Perivascular fat, a specialized adipose depot, plays a crucial role in vascular inflammation and remodeling by releasing a diverse array of bioactive molecules that exert both endocrine and paracrine effects on the vascular wall [6]. The communication between adipose tissue and the vascular wall is bidirectional [7, 8], indicating that the biological characteristics of adipose tissue are closely related to vascular lesions. The peri-coronary fat attenuation index (FAI), evaluated through standard coronary CTA, has emerged as a novel imaging biomarker for coronary inflammation, providing additional prognostic value for cardiovascular risk. [9, 10]. However, it remains uncertain whether aorta-specific FAI provides supplementary activity evaluation value for TA, without incurring additional costs or radiation exposure. The objective of this study was to utilize FAI to assess its efficacy in distinguishing TA patients with activity from those without.

## Materials and methods

### Study participants

**Fig. 1:**
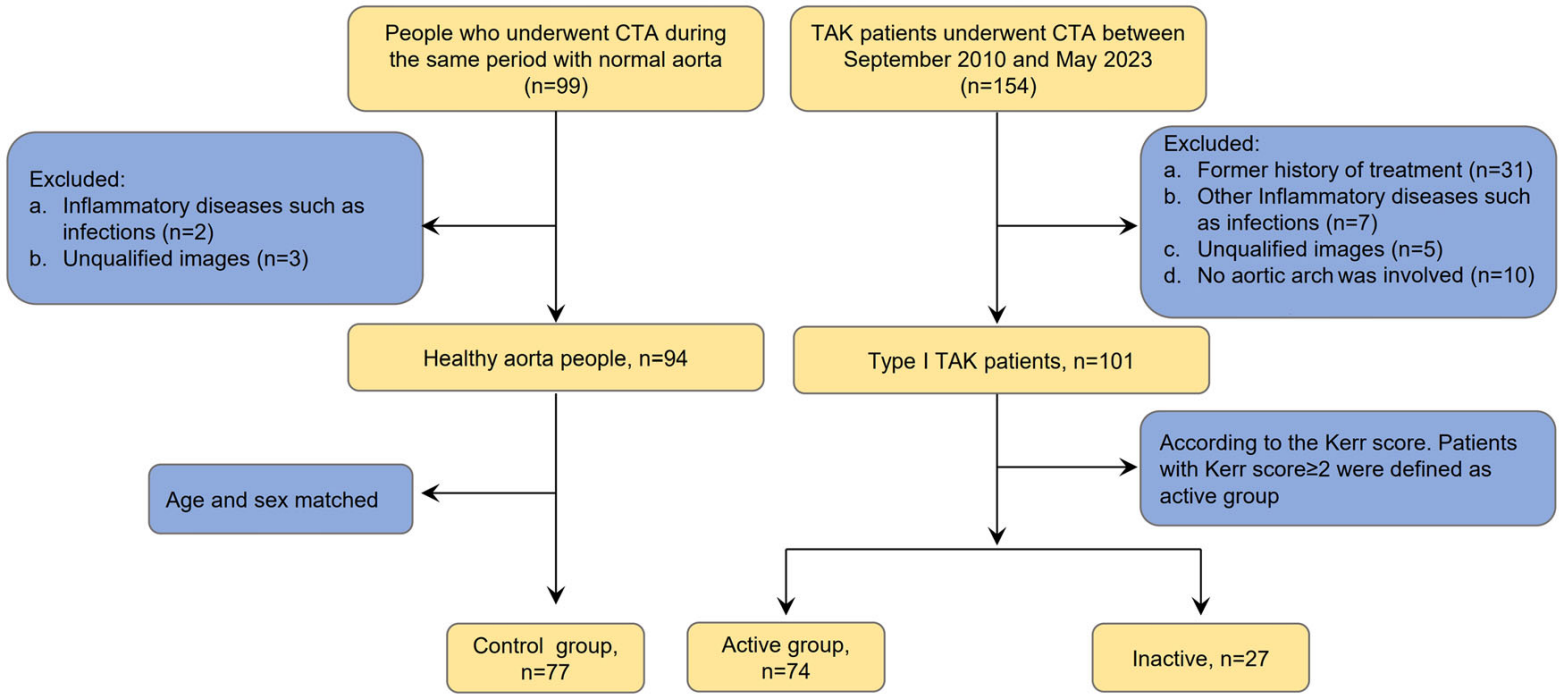
Study flow chat

A total of 154 consecutive patients were diagnosed as TA according to the 1990 American College of Rheumatology (ACR) Classification Criteria [11] from September 2015 to May 2023 in the first affiliated hospital of Xi’an Jiaotong University. Only patients with a new diagnosis of TA who were treatment-naïve at the time of their CTA were included in the study cohort to ensure an unconfounded assessment of baseline peri-aortic FAI. Of these, 53 were excluded due to prior treatment (*n* = 31), other inflammatory diseases (*n* = 7), poor-quality images (*n* = 5), or lack of aortic arch involvement (*n* = 10). Thus, 101 patients were included in the study. Meanwhile, we reviewed patients who underwent aorta CTA exams for chest pain, dyspnea, palpitation, or related symptoms at the same site and time. The control group (*n* = 77) was frequency-matched to the overall TA cohort based on age and gender to ensure comparable distributions. Figure 1 shows the patient recruitment and study design flowchart. The study was approved by the ethics committee of the First Affiliated Hospital of Xi’an Jiaotong University (No. XJTU1AF2021LSK-357), and the requirement for written informed consent was waived due to the retrospective analysis of the study and did not access or disclose personally identifiable patient information. We recorded patients’ clinical characteristics, age at CTA, geographic origin, sex, cardiovascular risk factors, associated diseases, blood tests, CRP levels, ESR, and imaging features.

### Disease activity evaluation

The Kerr criteria served as the benchmark for assessing disease activity, encompassing the following parameters: (i) systemic symptoms, with exclusions for infections and tumors; (ii) elevated ESR levels; (iii) vascular ischemic symptoms or signs, such as diminished or absent pulse, vascular bruits, or asymmetrical blood pressure; and (iv) positive imaging findings. The emergence or exacerbation of two or more of these criteria was indicative of “active disease” [3].

### Aortic CTA acquisition

All study patients had aortic CTA scans using two 256-row CT scanners: Philips Brilliance iCT and GE Revolution CT. An ECG trace was recorded. Iohexol (350 mg/mL) was injected at 2.0 mL/kg and 3.0 mL/s. Scanning started when the aortic arch’s CT value reached 150 HU, covering from the lower neck to the thorax’s base with the patient supine. Further details are in Supplementary Table S1.

### Analysis of CTA images

Morphological measurements from CTA were analyzed using a standard workstation (uInnovation-CT, R001, United Imaging Healthcare) via multiplanar reconstruction perpendicular to the adjusted aortic centerline. The study focused on the aortic arch and neck vessels, including the brachiocephalic trunk, subclavian, and common carotid arteries. CT images were assessed for: 1) Maximal wall thickness of affected vascular, measured independently by two observers using arterial phase images; 2) Number of branches involved; 3) Luminal abnormalities, including stenosis, occlusion, dilation, or aneurysm, with stenosis graded as mild (1–49%), moderate (50–70%), severe (70–99%), and occlusion (100%) [12]; 4) Standardized CT value of the aortic wall: measured CT attenuation on post-contrast images using a circular ROI, matching the area used for maximal wall thickness measurement. The ROI size was manually set based on arterial wall thickness. A 0.2 cm² circular ROI on the same image measured aortic lumen attenuation [13]. The standardized CT value of the aortic wall was defined as the ratio of mural attenuation to aortic lumen attenuation. 5) The presence or absence of side branches of collateral branches.

### FAI analysis

**Fig. 2:**
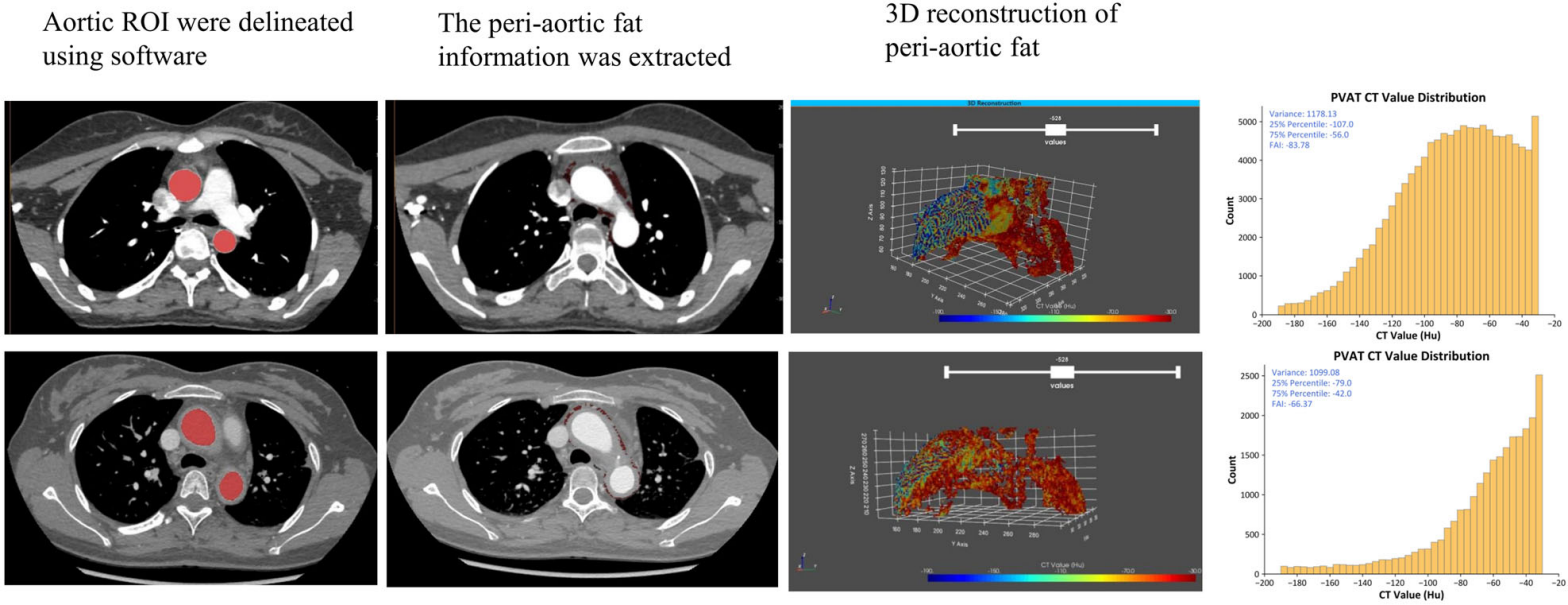
Evaluation indicators—FAI measurement. Examples of FAI measurement in control and TA patients

Lesion-based FAI was assessed using semi-automatic software (Version 1.0, United Imaging Research, Shanghai, China), which has been validated in previous studies for perivascular adipose tissue (PVAT) analysis [14]. Regions of interest were manually outlined by experienced radiologists to include lesion cross-sections, while avoiding adjacent normal tissues. PVAT was then automatically segmented based on CT values (−190 to −30 HU), and the software calculated PVAT attenuation by averaging fat pixels within 5 mm of the vessel’s outer wall (Fig. 2). The longitudinal range for PVAT attenuation matched the lesion’s boundaries, while the transverse range extended 5 mm around the aortic wall. Utilizing the perivascular fat attenuation histogram, the weighted attenuation value of perivascular fat was quantified after adjusting the technical parameters, resulting in the automatic calculation of the FAI.

Two radiologists, Hui Zhang and Zhijie Jian, with 10 and 15 years of vascular imaging experience, respectively, independently reviewed all images without access to clinical information. Disagreements were resolved by consensus, and average measurements were used for analysis.

### Reproducibility analysis

To evaluate intra- and inter-observer variability, two blinded investigators independently analyzed CTA parameters and FAI in a random sample of 30 participants. Variability was assessed using intra- and inter-class correlation coefficients (ICC) and their 95% confidence intervals (CIs).

### Statistical analysis

The data were analyzed using SPSS 25.0 (SPSS, Inc.) and R version 4.0.2 software. Continuous variables with normal distribution were expressed as mean ± standard deviation, and data with non-normal distribution were expressed as median with 25% and 75% interquartile range. Categorical variables are presented as cases (*n*) and percentages (count (%)). The distribution of the normality of continuous variables was examined using the Kolmogorov–Smirnov test. Normally distributed variables were compared using the independent samples *t*-test. Non-normally distributed variables were compared using the Mann-Whitney U test between two groups. Univariate and multivariate linear regression models were built to explore the relationship between FAI parameters and the markers of disease activity; logistic regression models were built to explore the relationship between FAI parameters and the diagnosis of disease activity of TA. The diagnostic value of FAI in the activity of TA patients was determined using the AUC of the ROC curve. A two-tailed probability (*p*) value < 0.05.

## Results

### Patient characteristics

The final analysis included 178 subjects: 101 with TA (average age 31.56 ± 9.52 years) and 77 without TA (average age 31.08 ± 9.61 years). Among TA patients, 89 were female with a median age of 30 years (IQR 23.0–37.0). Active TA patients showed higher platelet count, FAI, ESR, aortic wall thickness, Kerr score, affected branches, and CRP positivity, but lower HDL levels compared to inactive TA patients (Tables 1 and 2). TA and control groups were similar in age (median 30 vs. 31 years) and sex distribution (female, 88.1% vs. 87.0%). TA patients had lower cholesterol and triglycerides, but higher FAI, platelet and lymphocyte counts, and increased rates of anemia and osteoporosis (Supplementary Table S2).

**Table 1:** Baseline characteristics of individuals according to TA status

	Inactive (*n* = 27)	Active (*n* = 74)	*p*-value
FAI, HU	−80.53 (−83.72 to −75.73)	−75.51 (−78.31 to −72.15)	0.002
Age, years	33.00 (27.00–41.00)	29.50 (23.00–34.75)	0.161
Cholesterol, mmol/L	3.57 (3.04–3.86)	3.48 (3.01–4.28)	0.894
Triglyceride, mmol/L	0.98 (0.64–1.28)	0.78 (0.60–1.01)	0.170
LDL, mmol/L	1.91 (1.42–2.38)	2.03 (1.60–2.66)	0.247
HDL, mmol/L	1.17 (1.06–1.25)	0.96 (0.77–1.24)	0.010
Platelet, 10^9/L^3^	245.00 (210.50–299.50)	307.50 (224.00–387.00)	0.029
Lymphocyte, 10^9/L	1.90 (1.61–2.18)	1.96 (1.46–2.57)	0.994
Neutrophils, 10^9/L	4.06 (2.90–5.60)	4.68 (3.63–5.88)	0.167
White blood cell, 10^9/L^3^	6.39 (5.14–8.98)	7.40 (6.01–9.14)	0.186
Female, %	23 (85.19%)	66 (89.19%)	0.582
Hypertension, %	5 (18.52%)	17 (23.29%)	0.609
Anemia, %	1 (3.70%)	12 (16.44%)	0.093
Osteoporosis, %	3 (11.11%)	8 (10.96%)	0.983
Hyperlipidemia, %	0 (0.00%)	3 (4.11%)	0.285

*FAI* fat attenuation index, *LDL* Low-density lipoprotein, *HDL* high-density lipoprotein, *TA* Takayasu arteritis

**Table 2:** Imaging and clinical characteristics of individuals according to TA activity status

	Inactive (*n* = 27)	Active (=74)	*p*-value
FAI, HU	−80.53 (−83.72 to −75.73)	−75.51 (−78.31 to −72.15)	0.002
Aortic wall thickness, mm	3.50 (2.81–3.94)	4.18 (3.44–5.02)	0.004
Thickness of the affected vascular wall, mm	3.40 (2.67–4.67)	4.00 (3.07–5.28)	0.245
Number of affected branches, *n*	3.00 (1.50–3.00)	3.00 (3.00–3.00)	0.016
ESR, mm/h	13.00 (6.25–22.00)	35.50 (22.00–71.50)	< 0.001
Kerr score	1.00 (0.50–1.00)	3.00 (3.00–4.00)	< 0.001
CRP status	4 (14.81%)	43 (58.90%)	< 0.001
Severe stenosis of affected branches			0.614
1	12 (44.44%)	27 (36.49%)	
2	3 (11.11%)	17 (22.97%)	
3	6 (22.22%)	15 (20.27%)	
4	6 (22.22%)	15 (20.27%)	
Dilation or aneurysm, %	2 (7.41%)	8 (10.81%)	0.612
Lateral branch, %	6 (22.22%)	6 (8.11%)	0.052
Ring sign, %	10 (37.04%)	32 (43.24%)	0.575

### Distribution of aortic-FAI

**Fig. 3:**
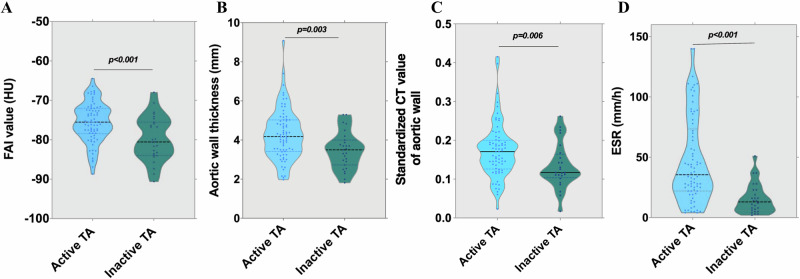
Distribution of clinical and imaging markers by disease activity. **A** Violin plot showing the distribution of the FAI value. **B** Aortic wall thickness. **C** Standardized CT value of aortic wall. **D** ESR according to the activity status of TA. HU, Hounsfield unit; FAI, fat attenuation index; ESR, erythrocyte sedimentation rate; TA, Takayasu arteritis

Aortic-FAI varied across study groups, with higher FAI value (*p* < 0.05) in active TA (−75.51, −78.31 to −72.15 HU) than inactive TA group (−80.53, −83.72 to −75.73 HU) (Fig. 3A). Compared with the inactive TA group, participants with active TA exhibited significantly greater aortic wall thickness, standardized CT value of aortic wall and ESR (Fig. 3B–D, all *p* < 0.05). Moreover, FAI value was higher in TA patients (−76.30, −80.41 to −72.67HU) than controls (−80.20, −84.41 to −76.98HU) (Fig. S1). There was no difference in aortic-FAI between controls and inactive TA group (Fig. S1).

### Diagnostic accuracy of aortic-FAI for TA and TA activity status

**Fig. 4:**
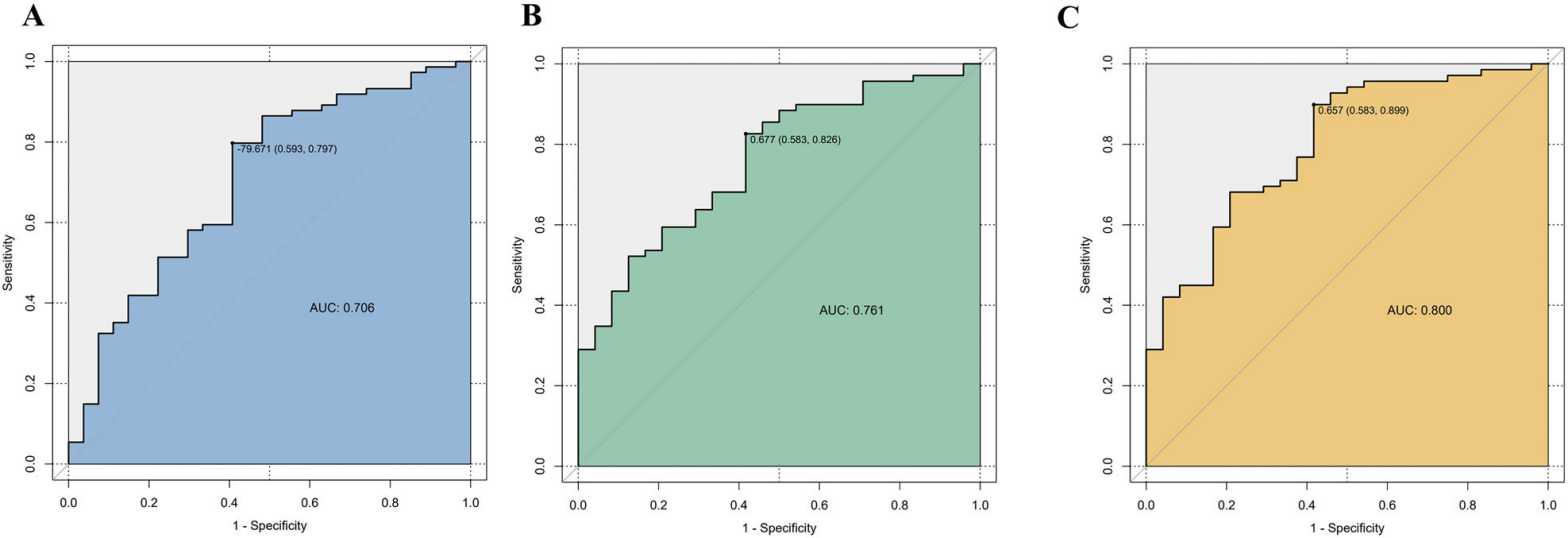
Diagnostic accuracy of FAI in the diagnosis and activity of TA. **A** Receiver operating characteristic curve of the FAI in activity of TA. **B** Model 1: aortic wall thickness and standardized CT value of aortic wall and number of affected branches; **C** FAI combined with Model 1. AUC, area under the curve

Aortic-FAI had good accuracy for differentiating TA patients with activity from inactive on ROC analysis (Fig. 4), and the AUC value was 0.71 (*p* = 0.002). Alone, aorta-specific FAI above −79 HU had 80% sensitivity and 60% specificity for differentiating active TA from all TA patients. The AUC was elevated when combined with other significant imaging factors, including aortic wall thickness, standardized CT value of aortic wall and number of affected branches (AUC = 0.80, *p* < 0.001), with 90% sensitivity and 58% specificity. Alone, aorta-specific FAI above −78 HU had 67% sensitivity and 68% specificity for differentiating TA from whole subjects, and the AUC value was 0.69 (*p* < 0.001) (Fig. S2).

### Association between aortic-FAI and the markers of disease activity

**Fig. 5:**
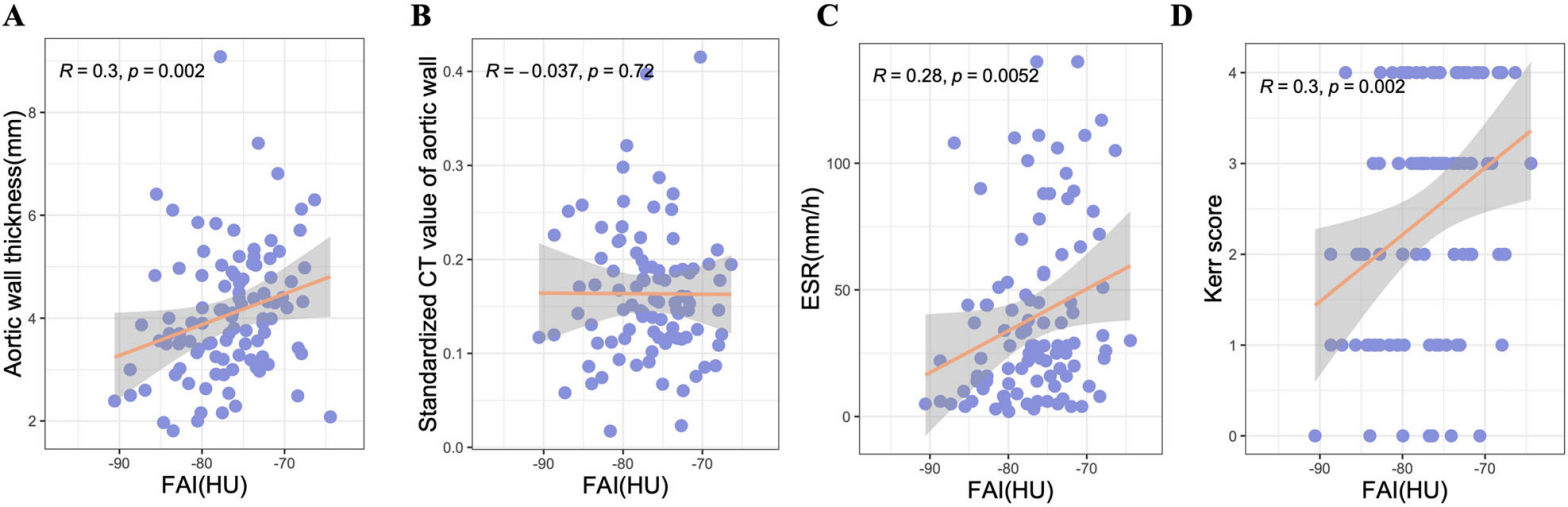
The regression line of the aortic FAI with the clinical and imaging markers of disease activity. **A** The regression line of the aortic FAI with aortic wall thickness. **B** Standardized CT value of the aortic wall. **C** ESR and (**D**) Kerr score. Plots showing Spearman’s correlations between FAI and clinical and imaging markers of disease activity. Abbreviations as Fig. 3

We further observed linear and positive associations of the aortic-FAI with the Kerr score (*r* = 0.304, *p* = 0.002), ESR (*r* = 0.278, *p* = 0.005) and aortic wall thickness (*r* = 0.305, *p* = 0.002), but not with standardized CT value of aortic wall, as shown in Fig. 5. The thickness of the affected vascular wall, number of affected branches and severity stenosis of affected branches were not associated with aortic-FAI. The correlation coefficients of the aortic-FAI with the markers of disease activity are presented in Supplementary Table S3.

In univariate analysis, a one-unit increase in the aortic-FAI was associated with a 0.07, 0.06 mm, and 1.65 mm/h increase in Kerr score, aortic wall thickness and ESR, respectively (Table 3). In the fully adjusted model (model 2), a one-unit increase in the aortic-FAI was associated with a 0.06 (95% CI): 0.01 to 0.10, *p* = 0.011) and 0.04 mm (95% CI: 0.002 to 0.081, *p* = 0.043) increase in Kerr score and aortic wall thickness, respectively, but not ESR (*β* = 0.67, 95% CI: −0.31 to 1.65, *p* = 0.182), as shown in Table 3. In logistic regression, FAI was a risk factor for active TA (OR = 1.16, 95% CI: 1.06 to 1.27, *p* = 0.002; OR = 1.14, 95% CI: 1.02 to 1.28, *p* = 0.025 for the fully adjusted model).

**Table 3:** Univariable and multivariable regression analysis between FAI and Kerr score, aortic wall thickness, ESR and TA activity status

	*β* (95% CI), *p*-value			OR (95% CI), *p*-value
Statistics	Kerr score	Aortic wall thickness (mm)	ESR (mm/h)	Active TA
Unadjusted	0.07 (0.03, 0.12), < 0.001	0.06 (0.02, 0.10), 0.008	1.65 (0.45, 2.85), 0.008	1.16 (1.06, 1.27), 0.002
Model 1	0.07 (0.02, 0.11), 0.004	0.08 (0.03, 0.12), < 0.001	1.49 (0.23, 2.75), 0.022	1.16 (1.05, 1.27), 0.003
Model 2	0.06 (0.01, 0.10) 0.011	0.04 (0.002, 0.081) 0.043	0.67 (−0.31, 1.65) 0.182	1.14 (1.02, 1.28) 0.025

Model 1: adjust for age, sex

Model 2: adjust for age, sex, CRP, cholesterol, platelets, hypertension and number of affected branches

### Intra- and inter-observer variability

Intra-observer and inter-observer variability of CTA parameters and FAI were shown in Supplemental Table S4. All the measurements showed excellent reproducibility, with ICC ranging from 0.822 to 0.916 in intra-observer and from 0.808 to 0.916 in inter-observer, respectively.

## Discussion

The accurate assessment of disease activity in patients with TA is crucial for determining appropriate treatment strategies and prognostic outcomes. The aorta and its major branches are primary targets in TAK. Therefore, in evaluating disease activity, it is preferable to seek non-invasive and highly cost-effective imaging evidence that can reflect the progression or resolution of vascular inflammation, in addition to identifying a variety of clinical blood markers. In our study, we found that aorta-specific FAI, derived from aortic CTA, was positively correlated with Kerr score and ESR. The combined assessment of FAI and the morphological features of the aorta on CTA may be useful for the differential diagnosis of disease activity. Both qualitative and quantitative insights into disease activity can be obtained without the need for additional examinations, which is beneficial for clinical management and efficacy assessment.

TA is a rare inflammatory disease of large arteries. The pathologic findings of TA demonstrate neoangiogenesis, macrophage and lymphocyte infiltration, arterial wall edema, degeneration of smooth muscle and elastic components, fibrosis and proliferation of fibroblasts and myofibroblasts, leading to wall thickening and lumen stenosis or dilation [15, 16].CTA has been widely used in the imaging of the vessel wall and lumen and the assessment of the extent of vessels involved in TA due to its advantages of high time and high spatial resolution [17, 18]. However, the efficiency of CTA in the assessment of disease activity remains to be further studied and practiced [19]. Found by Chen et al, maximal wall thickness and relative post-contrast enhancement ratio have a high sensitivity and specificity for detecting TA activity [13]. Our findings indicate that morphological and enhancement data obtained from CTA, specifically, vessel wall thickness, the degree of vessel wall enhancement, and the extent of branch involvement, are significant determinants of disease activity in patients, achieving an AUC of 0.761. However, morphology alone does not appear to adequately reflect or quantify the degree of inflammation in TA lesions. FAI is a radiological parameter indicative of the physiological state of PVAT. An elevated coronary FAI is associated with active vascular inflammation and is regarded as an imaging biomarker capable of effectively stratifying patients with clinical cardiovascular disease [9, 20]. In our study, the aortic peri-lesion FAI values were significantly higher in active patients compared to both control and inactive patients. FAI demonstrated a positive correlation with the Kerr score and ESR, indicating that FAI is associated with the degree of lesion inflammation. Furthermore, incorporating aorta-specific FAI derived from aortic CTA into our model enhanced the AUC to 0.8 in the diagnostic assessment of disease activity. When FAI is combined with effective morphological evaluation, a single CTA examination might reflect the pathological features of aortic wall edema, inflammatory cell infiltration, and vessel wall thickening during the active stage of TA.

As a novel imaging biomarker, perivascular FAI was initially employed to capture coronary inflammation by mapping the spatial changes in perivascular fat attenuation observed on coronary CTA [10, 21]. It has been utilized to assess the risk of coronary heart disease, evaluate plaque stability, and predict future cardiovascular events [9, 22–24]. In addition to coronary atherosclerotic heart disease, perivascular FAI, an inflammatory marker, can be utilized to monitor the response to biotherapy in patients with moderate-to-severe psoriasis. [25]. Yu Du et al reported a case in which perivascular FAI was used to measure and characterize pericoronary vasculitis due to IgG4-related arteritis with effective monitoring of treatment response [26]. These findings broaden the application of FAI to non-coronary atherosclerotic lesions, suggesting that FAI is capable of detecting pericoronary inflammation in chronic inflammatory conditions. The FAI in the peri-aortic fat is elevated, which is associated with the instability or rupture of abdominal aortic aneurysms [27]. Additionally, the CT value of fat surrounding spontaneous carotid artery dissection is higher than that in non-dissection areas, and with effective treatment, the CT value of fat around the lesion decreases [28]. This evidence suggests that periarterial FAI is also associated with local vasculopathy.

Although the absolute FAI difference between active and inactive TA was approximately 5 HU in our study, this magnitude aligns with, and in some cases exceeds, changes reported in prior studies as clinically meaningful. For instance, in coronary artery disease, a 4-6 HU increase in pericoronary FAI was associated with a significant rise in cardiac mortality risk [9]. In psoriasis patients, ~4 HU reduction in FAI after biologic therapy reflected decreased coronary inflammation [25]. Therefore, our observed difference of ~5 HU is well within the range of differences that the field considers biologically plausible and clinically meaningful for a para-inflammatory biomarker measured in peri-vascular fat. It is worth noting that we also attempted to assess the diagnostic efficacy of using FAI to diagnose TA alone in the present study. The diagnostic AUC of FAI for patient detection was 0.69, indicating moderate accuracy; its diagnostic capability is inferior to its effectiveness in assessing disease activity. This discrepancy may arise from the observation that FAI levels in inactive patients do not significantly differ from those in the control group, which obscures the overall differences in FAI between patients and controls. Consequently, we propose that FAI is better suited for evaluating patient activity rather than serving as a diagnostic tool for the disease.

Our study utilized CTA for the assessment of peri-aortic FAI, and it is important to consider both the advantages and limitations of this modality in the context of TA. A key strength of CTA is its excellent spatial resolution, which allows for precise anatomical delineation of the vessel wall and the surrounding fat, thereby enabling accurate FAI measurement. It also provides a comprehensive evaluation of vascular complications, such as stenosis, aneurysms, and lateral branch, in a single examination. However, as evidenced by the exclusion of five patients due to technically inadequate studies, CTA is susceptible to artifacts from patient motion and suboptimal contrast timing, which can preclude reliable quantification. Furthermore, CTA involves exposure to ionizing radiation and iodinated contrast medium. When compared to 18F-FDG PET/CT, which is highly sensitive for detecting metabolically active inflammation and is often considered a reference standard for disease activity [29], CTA-based FAI provides a different and potentially complementary insight. While FDG-PET directly visualizes glycolytic activity within the inflamed vessel wall [30], FAI aims to quantify the paracrine inflammatory changes in the peri-vascular adipose tissue. Moreover, FDG-PET excels in its superior sensitivity for early, metabolically active inflammation and whole-body assessment. Nevertheless, it has lower spatial resolution, higher cost, limited availability, and exposes patients to a substantially higher radiation dose [29]. Future prospective, multi-modality studies might directly compare FAI with FDG-PET uptake in the same patient cohort are warranted to elucidate their respective and potentially synergistic roles in the comprehensive management of TA.

We fully acknowledge that ESR and CRP are fundamental, cost-effective, and widely available first-line tests in the management of TA. However, as our study and extensive clinical experience show, these markers have well-documented limitations. Their sensitivity and specificity for identifying active vascular inflammation are suboptimal. A significant proportion of patients with active disease confirmed by imaging can have normal ESR and CRP levels, leading to a dangerous underestimation of disease activity [31]. Conversely, these markers can be elevated due to concurrent non-vascular infections or other inflammatory conditions. This is precisely where we propose FAI may offer distinct utility. Its value is not as a replacement for ESR/CRP, but as a complementary, objective, and anatomically specific tool in diagnostically challenging scenarios. FAI provides a direct, localized measure of the inflammatory activity in the peri-aortic niche, which is the primary disease target.

This study has several limitations. First, this study was a single-center retrospective study with information bias and selection bias. Secondly, the absence of follow-up data in this study. This limitation arises from the retrospective design of the study and the challenges associated with obtaining consistent follow-up imaging results in rare disease cohorts. Our cross-sectional study provides the foundational evidence for the association between peri-aortic FAI and disease activity in TA. The logical and critical next step is a prospective, longitudinal study designed specifically to evaluate the utility of FAI in monitoring treatment response and predicting clinical relapse. We will maintain ongoing surveillance of this cohort and perform scheduled follow-ups. Third, those who did not exhibit aortic arch involvement were excluded to maintain consistency in the vascular segments studied. Given that the pathological characteristics of the affected vessels at various sites are similar, the aortic arch region is the primary site impacted by TA lesions. Investigating this region can yield valuable insights into the FAI surrounding the lesions and assist in diagnosing disease activity. Future validation efforts will encompass a broader range of patients and will include classification studies based on the sites of involvement. Additionally, we employed the NIH criteria to classify disease activity, other utilized clinical activity evaluation criteria, such as ITAS [32], were not included in this analysis. Therefore, the value of FAI under alternative classification criteria warrants further investigation.

## Conclusion

Aorta-specific FAI based on CTA can be utilized to assess the activity of TA, thereby providing additional value to the morphological diagnosis obtained from CTA. This straightforward and effective imaging method holds promise as a new tool to assist clinicians in their decision-making processes. Further research is essential to evaluate the potential clinical utility of this imaging technique in monitoring disease activity and response to therapy in TA.

## Supplementary information

ELECTRONIC SUPPLEMENTARY MATERIAL

## Data Availability

The datasets used and/or analyzed during the current study are available from the corresponding author on reasonable request.

## Abbreviations

AUC: Area under the curve
CI: Confidence interval
CRP: C-reactive protein
CTA: Computed tomographic angiography
ESR: Erythrocyte sedimentation rate
FAI: Fat attenuation index
HU: Hounsfield units
ICC: Intraclass correlation coefficient
OR: Odds ratio
PVAT: Perivascular adipose tissue
ROI: Region of interest
TA: Takayasu arteritis
