# Supplementary material for: Utilization of aorta-specific fat attenuation index in evaluating disease activity and inflammation in Takayasu arteritis
Source: Insights Imaging. 2026 Mar 30;17:82. doi: 10.1186/s13244-026-02237-0 (PMC13035962; doi:10.1186/s13244-026-02237-0)

Utilization of Aorta-specific Fat Attenuation Index in Evaluating Disease Activity and Inflammation in Takayasu Arteritis

ELECTRONIC SUPPLEMENTARY MATERIAL

Supplemental table S1. Parameters of aortic CTA scanning.

	Philips Brilliance iCT	GE Revolution CT
Matrix size	512×512	512×512
Slice thickness, mm	0.625	0.625
Pitch, mm	0.900	0.992
Tube voltage, kV（depended on the patient's weight）		
<70 kg	100	100
≥70kg	120	120
Tube current, mA（depended on the patient's weight）		
<70 kg	300	300
≥70kg	250	250

Supplemental table S2. Characteristics of patients with or without TA.

	Control(n=77)	Whole TA(n=101)	P-value
FAI, HU	-80.20 (-84.41--76.98)	-76.30 (-80.41--72.67)*	<0.001
Age, years	31.00 (26.00-35.00)	30.00 (23.00-37.00)	0.475
Cholesterol,mmol/l	4.50 (3.25-5.00)	3.49 (3.00-4.27)*	<0.001
Triglyceride,mmol/l	1.56 (0.86-2.19)	0.80 (0.62-1.21)*	<0.001
LDL,mmol/l	2.42 (1.60-3.10)	2.03 (1.56-2.60)	0.092
HDL,mmol/l	1.06 (0.88-1.29)	1.06 (0.86-1.24)	0.723
Platelet,10^3 cells/dL	227.00 (194.00-284.00)	286.00 (216.00-357.00)	<0.001
Lymphocyte,	1.66 (1.34-2.12)	1.96 (1.49-2.43)*	0.029
Neutrophils,	4.97 (3.71-6.76)	4.54 (3.46-5.78)	0.207
White blood cell, 10^3	7.36 (5.89-9.24)	7.32 (5.76-9.08)	0.744
Female,%	67 (87.01%)	89 (88.12%)	0.824
Hypertension,%	12 (15.79%)	22 (22.00%)	0.301
Anemia,%	3 (3.95%)	13 (13.00%)*	0.039
Osteoporosis,%	1 (1.32%)	11 (11.00%)*	0.012
Hyperlipidemia,%	5 (6.58%)	3 (3.00%)	0.259

Supplemental table S3. Correlations of FAI with following factors.

	Correlation	P-value
Kerr score	0.3041	0.0020
ESR, mm/h	0.2776	0.0052
Aortic wall thickness, mm	0.3045	0.0020
Thickness of the affected vascular wall, mm	0.1333	0.1838
Number of affected branches, n	0.1107	0.2702
Severity stenosis of affected branches	0.0335	0.7393
Standardized CT value of aortic wall	-0.0372	0.7230

Supplemental table 4 The Intra-class Correlation of Intra - and inter-observer reproducibility for the assessment of CTA parameters and FAI

	Intra-observer		Inter-observer	
	ICC	95% CI	ICC	95% CI
Maximal wall thickness, mm	0.916	0.849 to 0.954	0.851	0.751 to 0.913
Stenosis severity, %	0.905	0.827 to 0.947	0.826	0.705 to 0.900
Standardized CT value of the aortic wall,%	0.822	0.722 to 0.901	0.808	0.739 to 0.948
FAI,Hu	0.845	0.738 to 0.911	0.916	0.849 to 0.954

Figure S1. Distribution of FAI value of by disease group

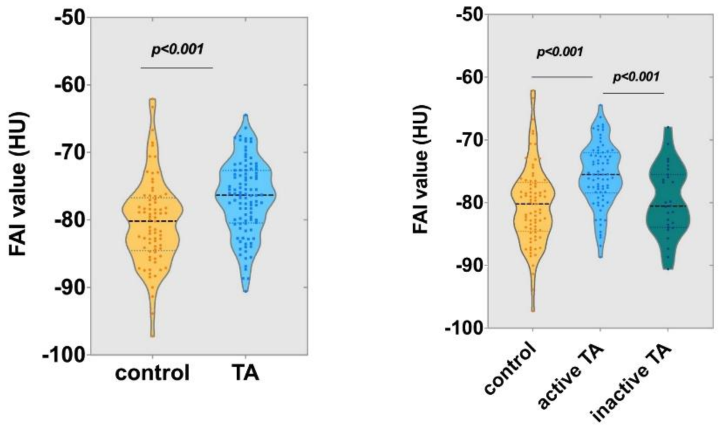

Figure S2. Receiver Operating Characteristic Curve of FAI in diagnosis of TA

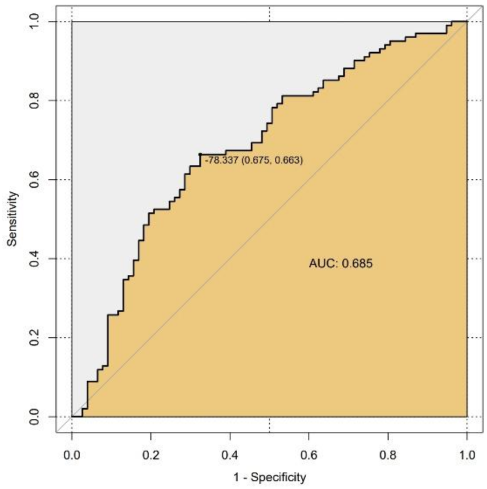

Supplement: Supplementary file 1 — ELECTRONIC SUPPLEMENTARY MATERIAL [file 13244_2026_2237_MOESM1_ESM.pdf]
